# Concomitant Frozen Elephant Trunk and Total Artificial Heart as a Bridge to Heart Transplantation

**DOI:** 10.1016/j.atssr.2024.07.022

**Published:** 2024-08-07

**Authors:** Orazio Amabile, Andrew Keogan, Modesto Colón, Mark Tasset, Ryan Ung, Ambar Andrade, Anantharam Kalya, Marc Silver, Radha Gopalan, Francisco Arabía

**Affiliations:** 1 Department of Surgery, University of Arizona, Phoenix, Arizona; 2 Department of Surgery, Creighton University, Phoenix, Arizona; 3 Department of Medicine, University of Arizona, Phoenix, Arizona

## Abstract

Patients with cardiomyopathy and aortic dissection presents significant challenges. Some patients are too ill to go directly to transplantation and require a bridge strategy to address the cardiomyopathy and dissection. This case combines a total artificial heart with a frozen elephant trunk procedure to increase the likelihood of survival. The patient underwent transposition of the left subclavian artery to the left carotid artery prior to frozen elephant trunk combined with implantation of a total artificial heart, bridge to transplantation. The patient remained inpatient, and underwent heart transplantation, with discharge from the hospital. Bridge to transplantation in patients with dissection is feasible in selected patients.

Stanford type A aortic dissections (ADs) are estimated to occur at an incidence of 8.7 per 100,00 patient-years, and those presenting at age <40 years are more likely to have Marfan syndrome (MS) or bicuspid aortic valve. Surgical management is well described in the American Association for Thoracic Surgery consensus document.^1^ Concomitant end-stage heart failure (ESHD) is rare in MS but represents a challenge in treating these patients in the setting of AD.^2^ Impaired cardiac function in MS has been attributed to valvular and nonvalvular nonischemic dysfunction, with heart transplantation (HT) well described. HT for severe heart failure after aortic arch replacement has been documented in cases of nonconnective tissue disorder AD^3^ and MS.^4^^,^^5^ However, bridge to transplantation (BTT) remains challenging in the setting of connective tissue disease and AD. The SynCardia Systems temporary (t-) total artificial heart (TAH) is a pneumatically driven biventricular replacement that is currently approved by the US Food and Drug Administration for BTT. The TAH implantation has been described in the medical literature. Surgical techniques to facilitate explantation have been described.^6^ Survival after bridging with t-TAH ranges from 71%-75% at 1 year. Survival reaches over 90% when the patient reaches transplantation.^7^^,^^8^

The patient presented with Marfan features (tall stature, arachnodactyly) and a maternal family history of thoracic AD. The patient demonstrated a Stanford type A dissection (intimal flap extending from coronary arteries to renal arteries) 6 days postpartum. She underwent a hemiarch replacement with aortic valve resuspension (bicuspid) under circulatory arrest. The patient presented 3 months postoperatively with acute decompensated heart failure with a left ventricle ejection fraction of 23%-30%; further evaluation was consistent with peripartum cardiomyopathy vs connective tissue disease cardiomyopathy. One year later, the patient was admitted for pneumonia and suffered a pulseless electrical activity arrest with subsequent placement of an internal cardiac defibrillator. She was readmitted, with ESHD (left ventricle ejection fraction, 10%), dependent on inotropic support. Computed tomography demonstrated a distal arch aneurysm of 4.15 cm with an intimal flap extending from the innominate and left subclavian arteries to the renal arteries with false lumen perfusion. The diameters of the axillary arteries were 3.6-4.0 mm. Echocardiography revealed severely reduced biventricular function. Right heart catheterization revealed right atrial pressure 16 mm Hg, pulmonary capillary wedge pressure 33 mmHg, and cardiac index 1.5 l/min/m^2^ on no inotropes. The patient was evaluated for HT but was at high risk for mortality if the dissection was not addressed. The patient was accepted for transplantation if the dissection and aneurysm could be repaired before transplantation. The severity of her biventricular failure and aortic dissection made it improbable for a successful bridge with a left ventricular assist device. The use of temporary percutaneous support as BTT in the presence of aortic dissection and small arteries disqualified her for this type of support. A TAH was chosen for BTT. In anticipation of TAH with frozen elephant trunk, she underwent left subclavian artery to carotid artery transposition 2 days prior without complication.

She was taken to the operating room for planned t-TAH, frozen elephant trunk, and automated implantable cardioverter defibrillator extraction. Initial plan for peripheral arterial cannulation was limited by diminutive peripheral arteries unsuitable for cannulation. After redo sternotomy, the heart, innominate, and common carotid vessels were dissected free from adhesions. Cannulation of the aortic arch, right superior vena cava, and right common femoral vein ensued with initiation of cardiopulmonary bypass and aortic cross-clamping. She was cooled to 24°C while ventriculectomies were performed. Atrial cuffs were sutured to atria at the mitral and tricuspid valve annuli levels. Once goal temperature had been reached, the innominate artery was cannulated, and antegrade cerebral perfusion was initiated; the innominate and common carotid arteries were clamped, and deep hypothermic circulatory arrest was initiated. The ascending aorta and arch just distal to the left common carotid artery were excised, and the aortic flap was fenestrated as distal as possible. A Thoraflex hybrid aortic graft (Terumo) was advanced into the aorta and deployed. The graft was sutured in a running fashion to the distal aorta. The perfusion limb was connected to the circuit, the 2 grafts were trimmed, and anastomoses were completed, running to the innominate and left common carotid artery. The graft was deaired, and a clamp was placed on the proximal portion of the new ascending graft. The patient was perfused, and rewarmed.

Circulatory arrest time was 27 minutes. Arterial conduits for the t-TAH were sutured to the pulmonary artery and new ascending aortic graft, and the drive lines were tunneled to the left upper quadrant of the abdomen. The t-TAH was connected to the atrial cuffs and arterial conduits. The system was deaired, and the patient was weaned from cardiopulmonary bypass after 2 hours 48 minutes. Circulation was now fully supported by the t-TAH (Figure 1).

**Figure 1:**
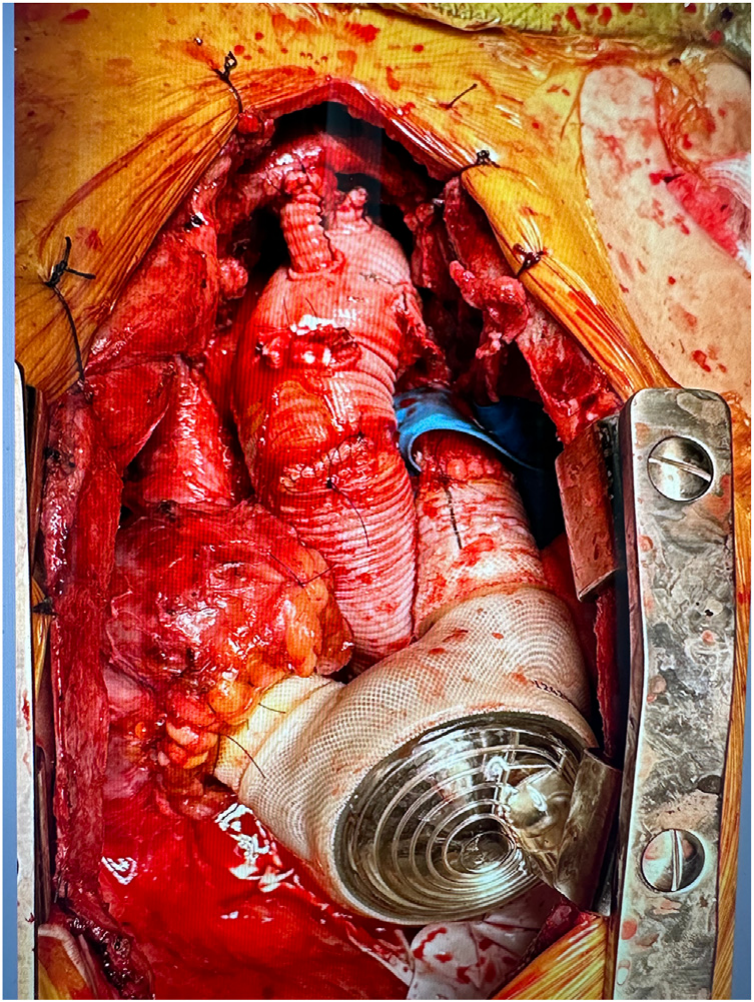
Temporary total artificial heart and frozen elephant trunk graft after completion.

The patient's chest was left open, and she recovered in the intensive care unit. On postoperative day 2, she returned to the operating room for l closure (Figure 2). Recovery was slow because of malnutrition and weakness. On postoperative day 76, she suffered a right caudate nucleus stroke without hemorrhagic conversion despite adequate anticoagulation with warfarin and aspirin. Computed tomography angiogram revealed a nonocclusive thrombus in the right M1 segment. She recovered neurologically and was listed as United Network for Organ Sharing (UNOS) status 2 for transplantation. The preoperative calculated panel reactive antibody was 29%, it increased to 99% but decreased to 77% at the time of transplantation. No desensitization was necessary and prospective crossmatch was negative. Renal function remained normal after TAH implantation and transplantation. No renal replacement therapy was required. The patient underwent HT on postoperative day 126 and was discharged to a rehabilitation facility and then home. The length of stay was 169 days. Although long, it was not a factor as the patient’s age was in the early 20s.

**Figure 2:**
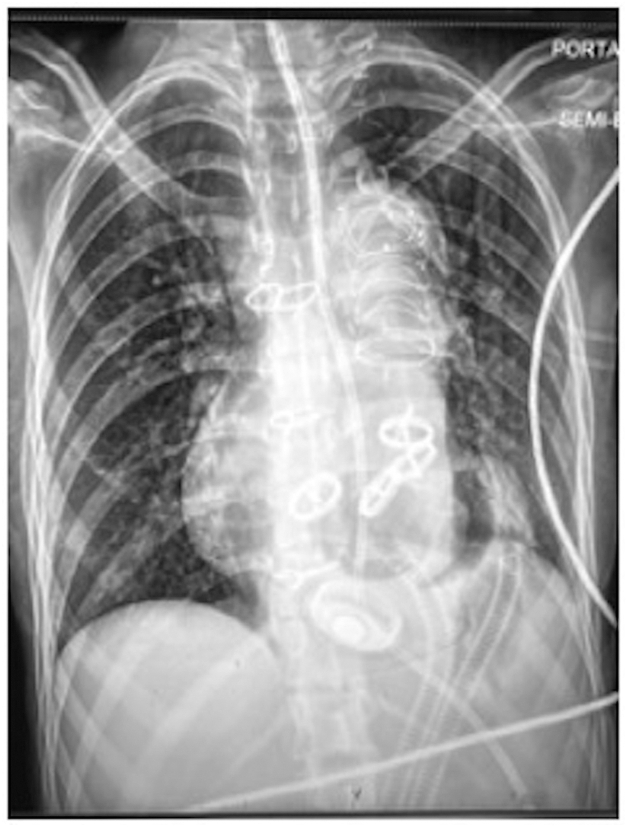
Chest radiograph showing temporary total artificial heart and frozen elephant trunk with distal aspect in the descending aorta.

## Comment

The approach represents a method for managing patients with Stanford type A dissection complicated by ESHD and connective tissue disorder who are eligible for HT. While this case demonstrated a favorable outcome, age and a relative absence of comorbidities contributed to its success. Transplantation in patients with MS has been previously studied, and long-term outcomes are comparable to non-Marfan syndrome patients. However, a challenge exists in bridging MS patients with extensive AD to transplant. Although the risk of the procedure appears to be significant, it seems less than temporary support followed by early transplantation or long-term support with a left ventricular assist device. The staging and combination of frozen elephant trunk with TAH in the appropriate patient for treating ESHD with concomitant Stanford type A dissections as a BTT may represent a reliable surgical pathway in the appropriately equipped center.
